# IFN-α Neutralizing Antibodies Distinguish LADA From Early-onset Type 1 Diabetes

**DOI:** 10.1210/clinem/dgaf001

**Published:** 2025-01-06

**Authors:** Rocco Amendolara, Luca D’Onofrio, Rosario Luigi Sessa, Stefano Di Giulio, Carmen Mignogna, Lucia Coraggio, Roberto Schirano, Simona Zampetti, Ilaria Malandrucco, Paolo Pozzilli, Giuseppe Giannini, Ernesto Maddaloni, Raffaella Buzzetti

**Affiliations:** Department of Experimental Medicine, Sapienza University of Rome, 00161 Rome, Italy; Department of Molecular Medicine, Sapienza University of Rome, 00161 Rome, Italy; Department of Experimental Medicine, Sapienza University of Rome, 00161 Rome, Italy; Department of Experimental Medicine, Sapienza University of Rome, 00161 Rome, Italy; Department of Molecular Medicine, Sapienza University of Rome, 00161 Rome, Italy; Department of Molecular Medicine, Sapienza University of Rome, 00161 Rome, Italy; Department of Experimental Medicine, Sapienza University of Rome, 00161 Rome, Italy; Department of Molecular Medicine, Sapienza University of Rome, 00161 Rome, Italy; Department of Experimental Medicine, Sapienza University of Rome, 00161 Rome, Italy; The UOSD of Endocrinology and Metabolic Diseases, Azienda Sanitaria Locale Frosinone, 03100 Frosinone, Italy; Department of Medicine, Unit of Endocrinology & Diabetes, Università Campus Bio-Medico di Roma, 00128 Rome, Italy; Centre of Immunobiology, Blizard Institute, St. Bartholomew's and London School of Medicine, London E1 2AT, UK; Department of Molecular Medicine, Sapienza University of Rome, 00161 Rome, Italy; Istituto Pasteur-Fondazione Cenci Bolognetti, 00161 Rome, Italy; Department of Experimental Medicine, Sapienza University of Rome, 00161 Rome, Italy; Department of Experimental Medicine, Sapienza University of Rome, 00161 Rome, Italy

**Keywords:** autoimmunity, LADA, latent autoimmune diabetes in adults, T1D, type 1 diabetes, IFN-α, neutralizing antibody

## Abstract

**Context:**

Autoantibodies against interferon-α (AAb-IFN-α) might be associated with the less aggressive autoimmunity in latent autoimmune diabetes in adults (LADA) compared to early-onset type 1 diabetes (T1D).

**Objective:**

To investigate the presence and clinical relevance of the positivity to AAb-IFN-α in people with LADA compared to T1D.

**Research Design and Methods:**

Serum levels of AAb-IFN-α isoforms were measured using a cell-based approach in 41 subjects with LADA and 90 subjects with T1D.

**Outcomes:**

The primary and secondary outcomes were the difference between LADA and T1D in the proportion of participants testing positive for autoantibodies (AAb) against ≥2 and against 3 interferon-α (IFN-α) isoforms, respectively. The presence and levels of AAb-IFN-α were related to clinical and biochemical features of participants with LADA.

**Results:**

Seven (17.1%) and 5 (12.2%) participants with LADA and 3 (3.3%) and 0 participants with T1D showed positivity for AAb against ≥2 and 3 IFN-α isoforms (*P* = .011 and *P* = .0025, respectively). Fasting blood glucose and hemoglobin A1c levels were numerically lower among people with LADA testing positive for AAb against ≥2 IFN-α isoforms than among those who were either negative or positive for AAb against 1 IFN-α isoform. Among LADA-positive individuals, levels of AAb-IFN-α2 isoform were inversely correlated with glutamic acid decarboxylase antibodies levels (rho = −0.513; *P* = .025).

**Conclusion:**

Autoimmunity against IFN-α is peculiar to autoimmune diabetes and appears to be distinctive to its slowly progressive forms. Understanding the underlying molecular mechanisms and clinical significance of this novel autoimmunity could lead to the development of new therapeutic strategies in autoimmune diseases, advancing personalized medicine.

Article Highlights

- IFN-α prompts the autoimmune attack against β-cells during the pathogenesis of T1D, and its endogenous neutralization may associate with milder forms of autoimmune diabetes (AD).
- Autoimmunity against IFN-α is peculiar to AD, representing a potential mechanism to distinguish LADA from early-onset T1D.
- Strong self-reactivity against IFN-α is associated with a better glycemic profile and lower glutamic acid decarboxylase antibodies levels in individuals with LADA.
- The existence of a natural autoimmunity against IFN-α may retain β-cells during the early stages of AD pathogenesis and over time, slowing the need for insulin treatment in people with slower progressive forms of AD.

Autoimmune diabetes (AD) is a heterogeneous autoimmune-driven disease ranging from more aggressive phenotypes characterized by a rapid loss of β-cell function, as observed in early-onset, “classic” type 1 diabetes (T1D), to milder forms, as latent autoimmune diabetes in adults (LADA). LADA is recognized as a slowly progressive form of AD characterized by a long-term preservation of endogenous insulin secretion capacity, despite the presence of pancreatic autoimmunity (1-4). Type 1 interferons, in particular interferon α (IFN-α), trigger the overexpression of human leukocyte antigen (HLA) class I (5), endoplasmic reticulum stress (6, 7) and β-cell apoptosis (8), ultimately resulting in progressive β-cell wasting (6). Human islets with residual β-cells from deceased subjects with T1D showed positivity for IFN-α and a concomitant hyperexpression of HLA I (9). The aberrant expression of HLA I may expose β-cell autoantigens to cytotoxic T-cells, spreading the autoimmune onslaught. In this context, the blockage of the IFN-α/β receptor with monoclonal antibodies prevents diabetes in nonobese diabetic mice (10, 11), and the inhibition of IFN-α intracellular signaling prevents the major histocompatibility complex (MHC) I overexpression in a model of β-cells (12). Recently, self-reactive antibodies targeting IFN-α have been associated with protection against T1D in people with mutations in the thymus transcription factor autoimmune regulator (13). No study has yet evaluated self-reactive autoantibodies against interferon-α (AAb-IFN-α) in a cohort of individuals with AD, including those with T1D and LADA. We hypothesize that individuals who develop strong self-reactions against IFN-α might experience a milder form of AD as this autoimmunity could potentially reduce β-cell exposure to immune-mediated cytotoxicity and subsequent cell death. Therefore, we conducted a cross-sectional study to primarily investigate the presence of this novel autoimmunity by comparing subjects with LADA to those with T1D. The study also aimed to explore whether and to what extent positivity to AAb-IFN-α is associated with a specific autoimmune disease phenotype. Specifically, the levels of AAb-IFN-α may be higher in individuals with LADA, who generally experience a slower decline in β-cell function compared to those with classic T1D.

## Research Design and Methods

### Study Design and Population

In this cross-sectional study, we enrolled participants with AD referring to the outpatient clinic of the Diabetology Unit of Policlinico Umberto I, General Hospital, Rome (Italy). Specifically, according to the sample size calculation, we measured AAb-IFN-α in sera collected from 41 subjects with LADA and 90 subjects with T1D. The cohort of people with T1D was made up of (1) a group of 49 subjects enrolled from a historical cohort for whom sera collected ≤12 months from T1D onset were available (14, 15) and (2) a group of 41 newly enrolled subjects with T1D matched 1:1 for sex and body mass index (BMI) (+/− 1 kg/m^2^) with LADA participants, among which 3 had a disease duration ≤12 months (Supplementary Fig. S1) (16). Such an enrollment strategy was chosen to allow the evaluation of differences in the primary outcome not only between LADA and T1D overall but also between (1) groups of people with LADA and T1D with comparable clinical features (Supplementary Table S1) (16) and (2) groups of people with T1D with different disease duration (recent vs no recent onset).

Study participants were carefully screened and enrolled according to the following inclusion criteria: (1) confirmed diagnosis of T1D, according to the American Diabetes Association guidelines (17), or confirmed diagnosis of LADA, according to the criteria proposed by the Immunology of Diabetes Society (18); (2) males and females aged 18 to 75 years; (3) ability and willingness to provide written and informed consent. Participants were excluded from this study if they had a diagnosis of other types of diabetes (monogenic diabetes), autoimmune polyendocrine syndrome type 1 (APS1), or systemic lupus erythematosus (SLE), chronic therapy with glucocorticoids, ongoing therapy with immunomodulators, pregnancy, liver cirrhosis, or any neoplasia in the last 5 years.

In addition, the levels of AAb-IFN-α were measured in the sera of a cohort of subjects with type 2 diabetes (T2D; n = 33), as a control group of people with a nonautoimmune form of diabetes.

### Clinical and Biochemical Measurements

The following anthropometric data, past medical history, and biochemical data were retrieved from the clinical charts of participants with LADA and of the newly enrolled participants with T1D: sex, age, duration of diabetes, height, weight, age at AD onset, smoking habits (current smoker was defined as smoking at least 1 cigarette/day in the last year), fasting blood glucose, glycated hemoglobin, total cholesterol, triglycerides, high-density lipoprotein cholesterol, and creatinine. BMI was calculated as weight in kilograms divided by height in square meters (kg/m^2^). The low-density lipoprotein cholesterol concentrations were estimated with the use of Friedewald et al's formula (19). The estimated glomerular filtration rate was estimated using the CKD-EPI formula, according to Levey et al (20). Blood pressure was measured on the day of clinical examination. Participants were kept resting in a sitting position for at least 5 to 10 minutes in a fasted condition, avoiding tobacco use or the intake of coffee or any stimulating beverage before the evaluation. The average of 3 consecutive measurements was used for the analysis. Hypertension was defined as a systolic blood pressure ≥140 mmHg or a diastolic blood pressure ≥90 mmHg or any use of antihypertensive drugs. The intensive insulin treatment was intended as multiple (≥3) daily injections or continuous subcutaneous insulin infusion. All participants' data were recorded in an anonymized Excel spreadsheet.

### Cell-based Assay to Study IFN-α Neutralizing Capacity of Patients' Sera

Blood samples collected after an overnight fast (8-10 hours) and stored at −80 °C were used for the measurement of AAb-ifN-α and pancreatic autoimmunity (see next section). The IFN-α neutralizing levels of participants' sera were tested using the following reporter cells: HEK-Blue^TM^ IFN-α/β cells (InvivoGen, San Diego, CA, USA; catalog no. hkb-ifnabv2, RRID: CVCL_KT26) expressing alkaline phosphatase under the inducible ISG54 promoter after ISGF binding to the IFN-stimulated response elements in the promoter, as previously reported (21). The cell line was cultured in DMEM (Sigma-Aldrich, St. Louis, MO, USA), according to manufacturer’s instruction. A calibration curve was set up to properly estimate the circulating concentration of AAb-IFN-α able to neutralize IFN-α using the anti-human IFN-α-IgG (InvivoGen; catalog no. hifna-mab1-02, RRID: AB_3665049).

The antibodies directed against the following 3 isoforms of IFN-α were evaluated in sera of study participants: IFN-α1 (InvivoGen; catalog no. rcyc-hifna1), IFN-α2b (InvivoGen; catalog no. rcyc-hifna2b), IFN-α14 (PBL Assay Science, Piscataway, NJ, USA; catalog no. 11145). These isoforms were chosen for 2 reasons: (1) AAb directed against them were present robustly in APS1/autoimmune polyendocrinopathy-candidiasis-ectodermal dystrophy patients with coexisting T1D (13), and (2) they efficiently react with the same commercially available antibody. A minimum concentration exerting the maximum cellular response was used as steady concentration, as follows: IFN-α1 was used at a final concentration of 5 ng/mL, whereas both IFN-α2 and IFN-α14 were used at a final concentration of 1 ng/mL. Quanti-Blue^TM^ Solution (InvivoGen; catalog no. rep-qbs) was used to determine alkaline phosphatase in the cell culture supernatants after an overnight incubation (18-21 hours). OD were measured at 630 nm with a microplate ELISA reader.

The thresholds for positivity were determined from the 99th centile of apparently healthy subjects (n = 47) and corresponded to 63.7 ng/mL for IFN-α1, 2.5 ng/mL for IFN-α2, and 49.1 ng/mL for IFN-α14.

Since neutralizing AAb-IFN-α cross-react with many IFN-α subtypes in a cohort of people with SLE and are associated with significantly reduced disease activity (22), and since a finding of ≥2 islet AAb usually confirms the diagnosis of adult-onset AD in apparent T2D without early insulin requirement (23), to exclude false positivity we considered that AAb against at least 2 IFN-α isoforms had to be present to confirm the presence of anti-IFN-α autoimmunity.

### Assessment of Pancreatic Autoimmunity

The serum concentration of glutamic acid decarboxylase antibodies (GADA) 65 and insulinoma-associated protein 2 antibodies (IA-2A) were measured using an immunoradiometric assay (Medipan GmbH, Blankenfelde-Mahlow, Germany; catalog no. 2070) and ELISA (RSR Limited, Cardiff, UK; catalog no. IAE/96/2, RRID: AB_2910240) kits, respectively.

### Statistical Analysis and Sample Size Considerations

Continuous variables are presented as means ± SD or median [25^th^-75^th^ percentiles], based on the distribution. Categorical variables are expressed as frequencies and percentages. The distribution of variables was tested graphically and with the Shapiro–Wilk test. A data point was considered an outlier if it is more than 1.5 ⋅ interquartile range above the third quartile or below the first quartile. Unpaired Student's *t*-test or the Mann–Whitney test was used to evaluate differences in continuous variables between groups, as appropriate. Depending on the distribution of the data, either the Kruskall–Wallis test or 1-way ANOVA was used. The primary and secondary outcomes of this study were the difference between groups in the proportion of participants testing positive for AAb against ≥2 and 3 IFN-α isoforms, respectively. As additional analyses, we also evaluated: (1) the difference between the groups in the proportion of AAb-positive subjects against each isoform of IFN-α; (2) the comparison of the main clinical features between patients with LADA + multiple AAb-IFN-α positivity and the remaining LADA group (AAb-negative and AAb-positive against 1 isoform of IFN-α); and (3) the correlation between the levels of each IFN-α isoform and the main clinical parameters known to be related to autoimmunity. Based on the distribution, Pearson's or Spearman's analyses were fitted to evaluate the presence of a correlation between serum levels of AAb-IFN-α and the main clinical/biochemical data.

The sample size was calculated on the primary outcome. We hypothesized to find a frequency of AAb-IFN-α positivity ≈15% among people with LADA and, based on published data (13), of ≤1% among individuals with T1D. According to these frequencies, a sample size of 40 participants with LADA and 85 participants with T1D was required to provide the study with 90% power to detect the hypothesized difference in the prevalence of multiple positivity (≥2 AAb-IFN-α) between the 2 study groups at an α level of .05.

Two-sided tests at the .05 level of significance were used for all statistical comparisons. Standardized mean difference (SMD) was also calculated as a measure of the effect size for the comparison between LADA with ≥2 AAb-IFN-α and LADA with 1 or no AAb-IFN-α. Statistical analyses were performed using SPSS statistical software (version 27; SPSS, Chicago, IL), and GraphPad Prism (version 10.2.3) software was used for graphical representations.

### Ethics Approval and Consent to Participate

The study was performed according to the Declaration of Helsinki, and the study protocol was approved by the institution's Ethic Committee (Comitato Etico “Sapienza,” from the Umberto I “Sapienza” University Hospital, in Rome; Prot. no. 287/2020). All participants signed a written informed consent to participate in the study.

## Results

### Prevalence and Profile of AAb-IFN-α

**Figure 1.**
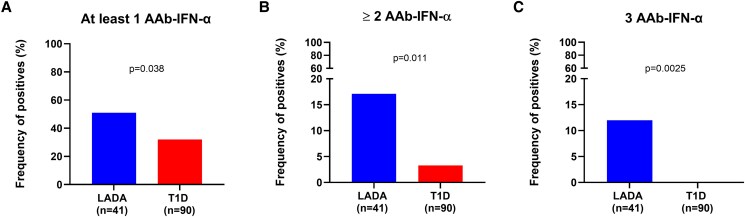
Frequency of AAb-IFN-α positivity in LADA vs T1D. Comparisons of the percentage of individuals with LADA (n = 41) and T1D (n = 90) who tested positive for (A) ≥ 1 AAb-IFN-α, (B) ≥ 2 AAb-IFN-α, or (C) all 3 AAb-IFN-α tested. Fisher's exact test or χ^2^ test was used to assess differences between study groups, as appropriate. Abbreviations: AAb-IFN-α, autoantibodies against interferon-α; LADA, latent autoimmune diabetes in adults; T1D, type 1 diabetes.

The prevalence and profile of AAb-IFN-α were compared between 41 subjects with LADA and 90 with T1D. A significantly higher proportion of participants testing positive for ≥2 AAb-IFN-α was found among subjects with LADA than among those with T1D [7 (17.1%) vs 3 (3.3%), *P* = .011; Fig. 1B]. All 3 subjects testing positive for ≥2 AAb-IFN-α had a recent-onset T1D (≤12 months). Five (12.2%) participants with LADA and none (0%) with T1D showed positivity to all 3 AAb-IFN-α tested (*P* = .0025; Fig. 1C). In addition, participants with LADA had a higher proportion of positives to at least 1 AAb-IFN-α [21 (52%) vs 29 (32%), *P* = .038); Fig. 1A]. Specifically, 6 (14.6%), 20 (48.8%), and 7 (17.1%) participants with LADA and 2 (2.2%), 21 (23.3%), and 10 (11.1%) of those with T1D tested positive for AAb-IFN-α1 (*P* = .012; Supplementary Fig. S2A) to AAb-IFN-α2 (*P* = .0036; Supplementary Fig. S2B) and to AAb-IFN-α14 (*P* = .40; Supplementary Fig. S2C), respectively (16).

All of the participants with T2D were negative for AAb against the 3 tested IFN-α isoforms (data not shown).

**Figure 2.**
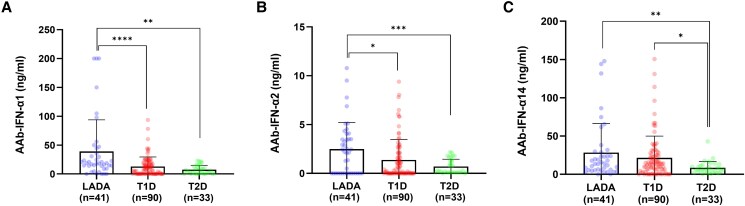
Serum neutralization capacity according to the type of diabetes. Comparison of AAb-IFN-α levels among subjects with LADA (n = 41), T1D (n = 90), and T2D (n = 33). The Mann–Whitney test was performed to assess differences between groups. Abbreviations: AAb-IFN-α, autoantibodies against interferon-α; LADA, latent autoimmune diabetes in adults; T1D, type 1 diabetes; T2D, type 2 diabetes.

The levels of AAb against IFN-α1 and IFN-α2 were significantly higher in people with LADA than those registered in people with T1D (*P* < .0001, *P* = .012, respectively) or T2D (*P* = .018, *P* < .0006, respectively), with no difference comparing subjects with T1D and T2D (AAb-IFN- α1: *P* = .32; AAb-IFN-α2: *P* = .63) (Fig. 2A-B). Levels of AAb against IFN-α14 were higher among people with LADA and T1D compared to T2D (*P* = .005 and *P* = .012, respectively), with no differences between LADA and T1D (*P* = .25) (Fig. 2C).

### Prevalence of AAb-IFN-α in Unrecent- vs Recent-Onset T1D

To avoid the possibility that the lower prevalence of AAb-IFN-α in the T1D cohort was due to long disease duration, we assessed the prevalence and profiles of AAb-IFN-α separately among people with T1D with a disease duration ≤12 months (n = 52) or >12 months (n = 38) (Supplementary Fig. S3A-E) (16). Three (5.6%) subjects with recent-onset T1D and none (0%) with longer T1D duration tested positive for ≥2 AAb-IFN-α (*P* = .26; Supplementary Fig. S3A) (16). People with recent and unrecent T1D did not also differ in terms of positivity to at least 1 AAb-IFN-α (45% vs 25%, *P* = .07; Supplementary Fig. S3B) (16). The prevalence of positivity against each isoform of IFN-α was similar in participants with recent-onset T1D when compared to people with longer T1D duration (*P* > .99 for AAb-IFN-α1, *P* = .28 for AAb-IFN-α2, and *P* = .73 for AAb-IFN-α14; Supplementary Fig. S3C-E) (16).

### Comparison of AAb-IFN-α Between People With LADA and T1D Matched for Sex and BMI

To compare autoimmunity against IFN-α in 2 groups of people with AD with comparable sex and BMI, we carried out analyses in the 41 participants with LADA compared to the 41 individuals with T1D, matched 1:1 for sex and BMI. Clinical, anthropometric, and biochemical features of the participants stratified by the type of diabetes are summarized in Supplementary Table S1 (16). Briefly, participants with LADA were older (*P* < .001), with a higher age at diagnosis (*P* < .001) and shorter duration of diabetes (*P* = .001); were less frequently smokers (*P* = .04); and had lower low-density lipoprotein cholesterol levels (*P* = .002) when compared to those with T1D (Supplementary Table S1) (16). At the time of enrollment, subjects with LADA required intensive insulin treatment less frequently than those with T1D (*P* < .001). No differences between groups are reported in terms of diabetes-related complications (data not shown).

Seven (17.1%) individuals with LADA and no (0%) patients with T1D tested positive for ≥2 AAb-IFN-α (*P* = .006; Supplementary Fig. S4A) (16). A numerically higher proportion of participants with LADA tested positive for AAb against each isoform (−1, −2, and −14) of IFN-α than those with T1D, although this was not statistically significant (Supplementary Fig. S4B-D) (16). In addition, participants with LADA positive for ≥2 AAb-IFN-α showed higher levels of AAb against each isoform of IFN-α (*P* = .006, *P* = .054, and *P* < .0001 for levels of AAb against the 1, 2, or 14 isoforms of IFN-α, respectively) when compared to single positives (Supplementary Fig. S5A-C) (16).

The overlap between antibodies against the different isoforms of IFN-α is shown in Supplementary Fig. S6 (16). Briefly, 5 patients with LADA were positive for 3 autoantibodies, 2 for 2 autoantibodies (1 with AAb-IFN-α1/AAb-IFN-α2 and 1 with AAb-IFN-α2/AAb-IFN-α14), and 14 for only 1 antibody (13 with AAb-IFN-α2 and 1 with AAb-IFN-α14).

### AAb-IFN-α Status and Clinical Phenotype in LADA

The clinical features of participants with LADA stratified by the AAb-IFN-α status are reported in Table 1. Participants with ≥2 AAb-IFN-α (n = 7) showed lower levels of fasting blood glucose (151.5 [124.8-219.3] vs 125 [92-162] mg/dL; *P* = .045, SMD: 0.88), numerically lower Hb1Ac (53 [43-63] vs 60 [50-66] mmol/mol; 7 [6.1-7.9] vs 7.6 [6.7-8.2] %; *P* = .37, SMD: 0.44), and higher levels of AAb against each isoform of IFN-α (*P* < .001 for all), when compared to the LADA group comprising individuals with 1 or no AAb-IFN-α (n = 34). No differences were observed between groups in terms of C-peptide, GADA, or IA-2A levels. In addition, among participants with LADA and ≥2 AAb-IFN-α, only 1 subject showed positivity for both GADA and IA-2A (*P* > .05). A numerically higher proportion of patients with LADA and ≥2 AAb-IFN-α was not on intensive insulin therapy compared to LADA patients with 1 or no AAb-IFN-α, although the difference was not statistically significant [3 (42.9%) vs 9 (26.5%), respectively; *P* = .66, SMD: 0.27].

**Table 1.** Clinical and biochemical characteristics of people with LADA stratified by the AAb-IFN-α status

	Negative/1 AAb (n = 34)	≥2 AAb (n = 7)	*P*-value	SMD
Age, years	53.5 [51-60.3]	55 [50-59]	.933	0.06
Males, n (%)	18 (53)	2 (29)	.410	0.48
Age at onset, years	46.5 [38-53.5]	43 [30-53]	.465	0.25
Duration of diabetes, years	6 [2.8-14.3]	10 [4-22]	.385	0.26
BMI, kg/m^2^	24.6 [22.3-29]	26.1 [24.5-27.3]	.314	0.03
HbA1c, %	7.6 [6.7-8.2]	7 [6.1-7.9]	.367	0.44
HbA1c, mmol/mol	60 [50-66]	53 [43-63]		
FBG, mg/dL	151.5 [124.8-219.3]	125 [92-162]	.045	0.88
Total cholesterol, mg/dL	174.8 (38.1)	186.3 (51.6)	.496	0.29
HDL-C, mg/dL	61.5 (17.5)	66.3 (21.7)	.536	0.26
LDL-C, mg/dL	78.5 [57.3-94.8]	63 [54-150]	.999	0.27
Tryglicerides, mg/dL	96.6 [73.5-116.3]	96.8 [69.4-109.6]	.747	0.01
Creatinine, mg/dL	0.8 [0.7-0.9]	0.6 [0.6-1]	.119	0.46
eGFR, mL/min/1.73m2	90 [76-102.5]	106 [83-120]	.088	0.72
C-peptide, ng/mL	0.6 [0.4-1.5]	1 [0.2-1.9]	.485	0.20
GADA, ng/mL	49.9 [4.61-68.6]	20.7 [0.7-81]	.578	0.18
IA-2A, U/mL	1 [0.7-16.3]	3 [0.6-4.6]	.906	0.42
AAb-IFN-α1, ng/mL	17.2 [9.2-23.7]	150 [98-200]	<.001	2.14
AAb-IFN-α2, ng/mL	1.3 [0-3.5]	6.9 [3-10]	<.001	1.74
AAb-IFN-α14, ng/mL	11.2 [4.9-20.6]	86.3 [65.5-144.1]	<.001	2.18
Intensive insulin treatment,*^a^* n (%)	22 (64.7)	4 (57)	.656	0.27
Smoking status, n (%)			.910	0.07
Current smoker	3 (10)	1 (14)		
Former smoker	6 (19)	1 (14)		
Never	22 (71)	5 (72)		
Hypertension, n (%)	10 (32)	3 (43)	.672	0.22
Thyroiditis, n (%)	12 (39)	3 (43)	.999	0.08

Abbreviations: AAb-IFN-α, autoantibodies against interferon-α; AAb-IFN-α, autoantibodies against interferon-α; BMI, body mass index; eGFR, estimated glomerular filtration rate; FBG, fasting blood glucose; GADA, glutamic acid decarboxylase antibodies; HbA1c: hemoglobin A1c; HDL-C, high-density lipoprotein cholesterol; IA-2A, insulinoma-associated protein 2 antibodies; LADA, latent autoimmune diabetes in adults; LDL-C, low-density lipoprotein cholesterol; SMD, standardized mean difference.

^*a*^Multiple daily injections or continuous subcutaneous insulin infusion.

**Figure 3.**
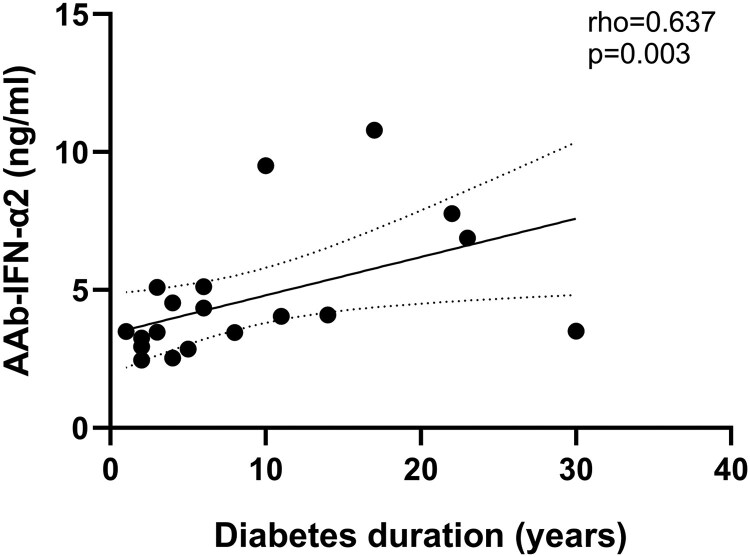
Correlation between AAb-IFN-α levels and diabetes duration. Correlation between levels of Aab-IFN-α isoform 2 and diabetes duration in individuals with LADA who tested positive for AAb-IFN-α2. Spearman's test was used to assess the monotonic covariation between these variables. Abbreviations: AAb-IFN-α, autoantibodies against interferon-α; LADA, latent autoimmune diabetes in adults.

**Figure 4.**
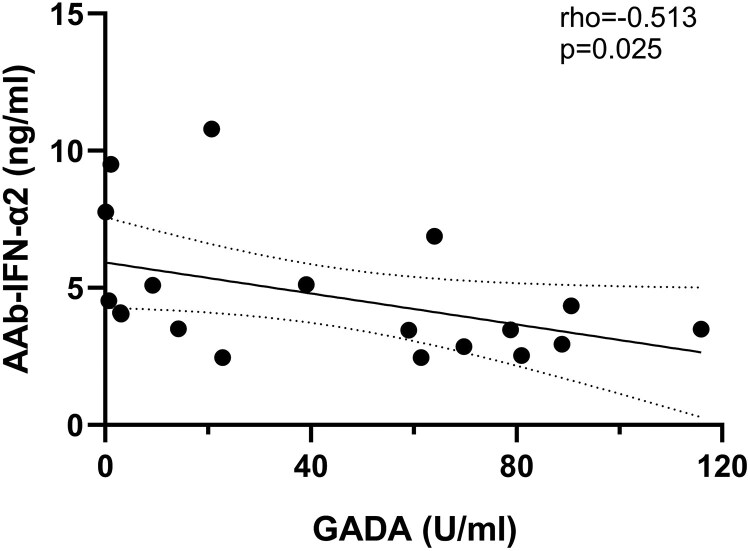
Correlation between AAb-IFN-α levels and GADA. Correlation between levels of AAb-IFN-α isoform 2b and GAD65 antibodies levels in individuals with LADA who tested positive for AAb-IFN-α2. Spearman's test was fitted to assess the monotonic covariation of measurements. Abbreviations: AAb-IFN-α, autoantibodies against interferon-α; GADA, glutamic acid decarboxylase antibodies; LADA, latent autoimmune diabetes in adults.

The levels of AAb against the IFN-α isoform 2 showed a positive correlation with diabetes duration (rho = 0.637, *P* = .003; Fig. 3) in LADA, while no correlation was found in T1D (rho −.220, *P* = .38, Supplementary Fig. S7) (16). In people with LADA, levels of AAb against the IFN-α isoform 2 were negatively related to GADA levels (rho = −.513, *P* = .025; Fig. 4). No correlation was found with other clinical and biochemical parameters.

## Discussion

In this study, we observed that a larger proportion of patients with LADA showed positivity for AAb neutralizing 2 or more isoforms of IFN-α, compared to early-onset T1D. Our data also suggest that strong self-reactivity against IFN-α is associated with better glycemic control and lower GADA levels among people with LADA. This evidence may indicate a potential protective role of AAb-IFN-α in slowing the decline of the β-cell reservoir in AD.

To the best of our knowledge, this is the first effort to unveil a novel autoimmunity to IFN-α epitopes and its possible clinical significance in a well-characterized cohort of people with different forms of AD and different profiles of disease progression. The harmful activity of IFN-α during the onset and progression of T1D has not yet been refuted. IFN-α is a cytokine, belonging to the type I IFNs, mainly involved in antiviral immune responses (24). Viral infections are a risk factor for the development of T1D, and the related IFN-α-driven antiviral response is considered a bridge between environmental exposure and the recruitment of immune cells to the pancreatic islets (25). Children genetically at risk for T1D show a type I IFN-inducible transcriptional signature in circulating cells, even preceding the development of islet AAb (26, 27), suggesting that IFN-α is mainly involved in the early stages of T1D and in the transition between innate and adaptive immune responses (6). IFN-α is a key driver of the β-cell inflammatory response during insulitis, inducing a gene signature that strongly correlates with the transcriptomic profile of β-cells from individuals with T1D, through a deep RNA sequencing approach (28). In addition, IFN-α upregulates the expression of endoplasmic reticulum stress mediators and the production of chemokines, leading to the spread of autoimmune reaction and β-cell reservoir decay (6). Type I IFNs also increase immune-mediated apoptosis of β-cells by enhancing the cytotoxicity of CD8+ T cells (29). Moreover, human islets with residual β-cells from deceased subjects with T1D showed strong positivity for IFN-α and a concurrent hyperexpression of class I MHC (9). Conversely, individuals with T2D and nonviral pancreatic inflammatory disorders failed to show the same positivity (9). In view of the involvement of IFN-α in the pathogenesis of AD, the endogenous neutralization of its disruptive signaling to prevent or delay the onset of AD is intriguing. The blockage of the IFN-α/β receptor with monoclonal antibodies prevents diabetes in nonobese diabetic mice (10, 11), and the inhibition of IFN-α-mediated intracellular signaling in a model of β-cells prevents the overexpression of MHC I (12). Furthermore, Janus kinase (JAK) inhibitors are able to halt the intracellular signaling of cytokines acting through the JAK–STAT pathway, including IFN-α. Baricitinib, a JAK1/2 inhibitor, protects human β-cells against cytokine-driven apoptosis and class I HLA overexpression (30). Interestingly, a recent phase II clinical trial demonstrated that baricitinib preserves insulin secretion after 48 weeks of treatment in patients with recent-onset T1D (31). Furthermore, Meyer et al reported that patients with APS1 and GADA positivity, which efficiently neutralized IFN-α activity, were protected against the onset of overt T1D (13). Likewise, inactive disease in people with SLE is linked to AAb against type I IFN, which normalizes blood levels of IFN-α and restores the normal function of B cell subsets (22). To date, the existence of a natural self-defense mechanism blocking the IFN-α response in settings of AD has never been assessed. Our data revealed that a simultaneous self-immune reaction against different isoforms of IFN-α is significantly more prevalent in individuals with LADA compared to those with T1D. This may represent an endogenous defensive response of the immune system against the aggressive autoimmune attack, typical of early-onset T1D, which leads to rapid β-cell destruction and the early need for insulin treatment at diagnosis. Individuals with LADA who have 2 or more antibodies against AAb-IFN-α appear to have a worse metabolic decompensation and are more frequently on multiple daily insulin injections. However, the small number of antibody-positive subjects limits the ability to draw definitive conclusions.

Our findings should be interpreted in the light of strengths and limitations. The major strength of the study has to be recognized in the novelty, providing evidence of the presence and clinical significance of this autoimmunity in a well-characterized cohort of patients with AD and shedding further light on AD pathogenesis. Our study included participants with different types of AD and disease duration, carefully screened and consecutively enrolled. Moreover, we assessed the presence of AAb-IFN-α in a cohort of people with T2D in order to rule out the possibility that other forms of diabetes could present such positivity. The lack of positivity in T2D suggests that autoimmunity against IFN-α is peculiar for AD and, in particular, appears to be distinctive of slowly progressing forms of AD. Although the primary outcome of our study was to evaluate the proportion of participants testing positive for AAb against ≥2 IFN-α isoforms, the complete absence of such AAb in people with T2D also decreases the possibility of false positivity, resulting in a stronger diagnostic value also of single positivity to AAb-IFN-α levels. According to a proper sample size evaluation, the study is adequately powered to discriminate differences in the frequency of multiple positivity between the study groups. In addition, the possibility of having false positives in the AAb-IFN-α test is limited by the choice to consider participants with AAb against at least 2 IFN-α isoforms as true positives. This is also confirmed by the fact that participants with ≥2 AAb-IFN-α also had higher AAb levels against each isoform of IFN-α. In addition, the measurement of AAb-IFN-α levels was performed in a cohort of people with recent-onset T1D to rule out the possibility that AAb-IFN-α levels decreased with increasing duration of diabetes, as occurs with pancreatic AAb (32, 33). Finally, the methodological approach used to measure AAb-IFN-α is able to assess the IFN-α-neutralizing ability of patients' sera, allowing us to consider only actually functioning AAb- IFN-α.

However, some limitations must be acknowledged. First, the cross-sectional design did not allow us to make any inference regarding causality. Second, the small number of participants positive for AAb against IFN-α1 and IFN-α14 prevents us from drawing solid conclusions about the association between anti-IFN-α autoimmunity and the clinical characteristics of patients with LADA. Lastly, the lack of clinical characteristics for the majority of participants in the historical cohort of recent-onset T1D hampered the assessment of differences between the groups in terms of clinical characteristics.

In conclusion, this study provides the first evidence of endogenous anti-IFN-α reactivity as an autoimmune response in LADA, a slowly progressive form of AD. This response is less commonly observed in people with the rapidly progressive form of the disease, and it is not present in people with T2D. Further large-scale and prospective studies are required to elucidate the clinical significance of this autoimmunity in the pathogenesis of AD and to assess the potential role of AAb-IFN-α as a protective self-immune response against the rapid decline of β-cell mass.

## Data Availability

Some or all datasets generated during and/or analyzed during the current study are not publicly available but are available from the corresponding author on reasonable request.

## Abbreviations

AAb: autoantibodies
AAb-IFN-α: autoantibodies against interferon-α
AD: autoimmune diabetes
APS-1: autoimmune polyendocrine syndrome type 1
BMI: body mass index
GADA: glutamic acid decarboxylase antibodies
HLA: human leukocyte antigen
IA-2A: insulinoma-associated protein 2 antibodies
IFN-α: interferon α
JAK: Janus kinase
LADA: latent autoimmune diabetes in adults
MHC: major histocompatibility complex
SLE: systemic lupus erythematosus
SME: standardized mean difference
T1D: type 1 diabetes
T2D: type 2 diabetes
